# Initiating count down - gamification of academic integrity

**DOI:** 10.1007/s40979-020-00068-0

**Published:** 2021-03-18

**Authors:** Zeenath Reza Khan, Jarret Dyer, Sonja Bjelobaba, Sandra F. Gomes, Dita Henek Dlabolová, Shivadas Sivasubramaniam, Soly Mathew Biju, Ajrina Hysaj, Priyanka Harish

**Affiliations:** 1 University of Wollongong in Dubai, Dubai, UAE; 2 College of DuPage, Glen Ellyn, USA; 3 Uppsala University, Uppsala, Sweden; 4 University of Porto, Porto, Portugal; 5 Mendel University, Brno, Czech Republic; 6 University of Derby, Derby, UK

**Keywords:** Gamification, Contract cheating, Academic integrity, Games, Proactive training

## Abstract

Any problem is a problem until a solution is designed and implemented. This paper reports on a workshop that highlights preliminary work done by the working group on Gamification in the scope of European Network for Academic Integrity (ENAI), which aims to explore the possibility of developing and testing a gamified learning module on academic integrity values. In this paper, the group aims to look at proposing steps we are currently using to develop storyboards of scenarios for the first phase of the project, which were presented at the 6th International Conference Plagiarism Across Europe and Beyond 2020 held virtually in Dubai as a workshop. The study also presents updated findings and scenarios drawn from the workshop conducted and audience feedback, in the following sections that pave the way for the future stages of the gamification process. This serves as a guide to academics and researchers in academic integrity who may wish to study gamification and apply it to develop their own modules for their learning modules.

## Introduction

In the twenty-first century, the academic world is facing new threats to maintain integrity. Commercialisation of education together with the rise in the use of online resources and its resultant ghost-writing services have impacted teaching and research (Evans, 2001; Kezar & Bernstein-Sierra, 2016). These external factors have forced the sector to think about innovative ways to teach, deliver and most importantly to engage students to “embrace” integrity. The traditional lecture-based delivery, which is mostly in the form of *furor loquendi*, from lecturer to students, is no longer practicable. The days of one-way flow of knowledge from the academics to the students are almost over. There is also an emphasis/expectation for the transformation of students’ attitude of “learning for assessments” into “learning for application”. In fact, modern pedagogy expects students to embrace both individual and societal values and apply their knowledge/skills in their future professions (Mynbayeva et al., 2017). The Glossary of Education Reform (Great Schools Partnerships, 2016) suggests student engagement is often dependent on the individual’s curiosity, interest, optimism, and passion but is influenced by how they are being taught, and whether the methodology of teaching enhances their motivation, critical thinking and attention.

We as academics are facing a dilemma of developing innovative teaching methods, not only for imparting knowledge but also in deterring academic misconducts amongst students. For this reason, different methodologies for student engagement are being developed and tested (López et al., 2013; Sivasubramaniam, 2013; Wahabi & Al-Ansary, 2011; Nageswari et al., 2004). Approaches such as enquiry based learning (which enhances students inquisitiveness); use of jigsaws (to enhance cooperative learning in groups); project based learning (where students’ address/solve challenges based on real-world scenarios) oBaumr debate-based learning (by creating interesting topics for students to argue and conclude). Furthermore, flipped learning (or SCALE-UP - Student-Centred Active Learning Environment with Upside-down Pedagogies) and formative “feed-forward” for reflective learning (engaging students with continuous feedback linked to assessment) are being used in higher education and gaining popularity amongst schools too. In addition, technology assisted and/or enhanced learning strategies such as the use of quick response (QR) codes (linking to information within the classroom settings); wisely managed classroom (where teaching is enhanced via individualised use of technology such as smartphone or tablet) (Kirkwood & Price 2014) are commonly used. Innovative teaching/learning strategy that has gained some popularity in recent years is the use of gamification and game-based learning which creates an indirect, yet life-long learning environment by enhancing participants’ natural desire to play games via socialising (Arnold, 2014).

Gamification is adding gaming elements such as points, rewards and other game mechanics while game-based learning is learning through games but both strategies are able to promote significant content mastery and sustained learning (TeachThought, 2019). In 2001, Prensky suggested game based learning (GBL) as a thought-provoking, adrenaline filled and high goal achieving learning process. GBL is often used to teach new concepts where the student’s progress in the game is linked to their understanding of the concepts taught while gamification drives learning outcomes (Prensky, 2001). Using GBL and gamification then allows for a complete solution that offers a game based on teaching material as a one-stop-shop.

The use and issues related to GBL and gamification in higher education have started being analysed extensively since 2012. In the start, GBL was used as pedagogical and cognitive tools to help with all the aspects of language acquisition (O'Rourke et al., 2013; Gee, 2008; Hayes & Games, 2008). Furthermore in 2012, Wu and Richards enumerated that digital games helped learners of English as a second language develop an array of language skills mainly related to literacy e.g. spelling, pronunciation reading, writing, sentence formation and punctuation (Wu & Richards, 2012). Recently, use of GBL and gamification have been reported in areas such as teaching sciences (Khan et al., 2017), to create elements of orientation for new students (Fitz-Walter et al., 2011), increase student engagement (Tan & Sockalingam, 2015) and so on.

Using this understanding of student learning and technology, researchers have posited that gamification of academic integrity (the other ‘AI’) can provide a solid ground for utilisation of fun during different stages of the learning process (Anderson et al., 2001; Bloom et al., 1956). Thus, the objective of this paper is to describe preliminary work done by the working group on Gamification in the scope of European Network for Academic Integrity (ENAI) aiming to develop a gamified learning module on contract cheating as the pilot phase of the group’s work.

## Academic misconduct and technology

Student cheating is not new because they are a part of a society which has shown evolutionary traits for cheating (Ghoul et al., 2013). That is cheating can be considered as one of the human behaviours where people attempt to circumvent the rules or find shortcuts to achieve goals. From Bowers (1964) to McCabe and Bowers (1994) and every researcher since, literature has captured numerous instances of self-reported student cheating cases because academics and researchers alike understand the importance of highlighting and speaking out against misconduct. Similarly, in 1999 a cover story in the US News and World report by Kleiner and Lord has claimed around 75% to 98% of all students admitted to having cheated at least once academic cheating; the article had a rightful title “‘Everyone’s doing it’, from grade school to graduate school” (Kleiner & Lord, 1999). As technology has infiltrated the world of academia giving rise to Smart Education teaching and learning environments, the challenges have become somewhat more complex and varied (Khan & Balasubramanian, 2012; Khan, 2014; Khan, 2019). Smart education is the incorporation of cutting edge technology to enhance student learning, where “[l] earners utilize smart devices to access digital resources through wireless network [s] and to immerse in both personalized and seamless learning” (Zhu et al., 2016; p1). Cyber plagiarism, for instance, has become an emerging issue in addition to other conventional types of academic misconduct (Eysenbach, 2000). Internet search engines such as Google® (including Google Scholar) have made it easier to search for any topics; so the availability of resources are at the tip of the fingers of any internet user. In fact, it is easy to find anything, whether it is scientific ideas or merely mythical information by a simple Google® search, making it easy to plagiarise. In contrast, the growth of technology has also helped us to enhance integrity. Digital tools have also been developed to detect misconduct and promote AI, such as mobile eye-tracking technology to identify cheating, especially during examination (Thomas and Jeffers, 2019). In fact, a wide range of tools for online test security was reported as early as 2009 (Hart and Morgan, 2009). According to Ye and Lin (2019) the technology based solutions (such as Adobe Photoshop® Droplets and Motunin®) can also be used to detect excessively (or improperly) manipulated images, a problem that has been known in STEM (Bik et al., 2016).

Another growing threat to AI, contract cheating was firstly described in 2006. It comprises an independent way of academic misconduct with a student’s work submission as result of a contracted and paid service (Clarke & Lancaster, 2006). With a prevalence of around 6% on post- and undergraduate levels, ghost-written work and fraudulent online test assessment are the most common misconduct practices reported by the students (Bretag, 2019). Earliest recorded cases show how fraternity houses used to keep essay mills in their basements and how they would encourage their members to recycle submitted essays (Singh & Remenyi, 2015). Morality and motivation for learning are the key factors reported by the students to not engage in contract cheating (Rundle et al., 2019). On the other hand, the difficulties associated with a second language, the availability of a wide range of opportunities to cheat and a propitious academic environment are elements considered as promoters of misconduct (Bretag, 2019). Irrespective of the type of misconduct, the validity of higher education assessments as a measure of learning is undermined. If students are engaging in misconduct, integrity of education is being threatened because it is considered the foundation of academia.

## Need for proactive measures to develop a culture of integrity

To help deter students from contract cheating and other forms of misconducts, recent studies have attempted to aid academics through researching areas such as legal approaches (Draper & Newton, 2017), detection (Rogerson, 2017), and analyzing the advertisements (Kaktiņš, 2018). However, we believe the focus needs to be more proactive, than reactive. In addition, the aims of these proactive measures should revolve around making students to become “active learners”. As Freeman et al. (2014) suggested students should take the centre stage in the learning process. By this way, they would become engaged and therefore embrace integrity as a part of that discourse. The literature surveys discussed herein also emphasise the importance of “making things interesting” to the learners. In addition to individual responsibility, the academic environment may influence student’s behaviour (Dyer et al., 2020). Similarly, in research practice, impact ranking, competition related pressures to maintain publication record, may impact upon integrity (Binder et al., 2016). At institutional level, ethics committees as well as conduct and ethical codes with associated penalties are two available strategies to enhance and support an AI environment as formalized in the 1980s to address efforts for an increased prevalence of misconduct and corruption cases in companies and public institutions. Therefore, there is a need to design, develop and deliver co-curricular modules to enhance integrity amongst students and researchers. One such method is to design and implement learning modules that train students on AI values and academic writing skills. The Quality Assurance Agency for Higher Education in the UK stated “Quality student information and support are central to any strategy aimed at encouraging academic integrity and reducing contract cheating. Providers can foster academic integrity through promoting scholarly institutional values, engaging in dialogue with the student community and ensuring that academic and professional staff are aware and aligned with a set of common aims and objectives” (Quality Assurance Agency, 2017).

A quick search on various university websites shows most modules on the topic related to AI focus on promoting studying, writing skills, communication skills, time management and so on. Studies do posit the importance of informing students about institutional policies which cover definitions of AI, however, often, there are no modules dedicated to imparting the values to students as a proactive approach. Moreover, a recent white-paper study on the effectiveness of training modules has shown the need to engage students beyond text-heavy, traditional teaching modules (Global Challenges UOWD-UOW Collaborative Project #2018-GC1-ZRSM). One way to make these modules more engaging perhaps lies in using technology itself. Digital disruptors have created waves in recent years in the education sector with tools such as gamification, artificial intelligence, augmented reality, virtual reality, big data and so on (Gardner, 2016). The next section looks at how this may be an avenue that can help sustain long-term learning and collaboration among students when teaching them academic integrity values.

## Gamification in education and AI

Digital disruption can be an intimidating phrase, however, it is a vital/necessary way of changing how things are done traditionally. Digital disruptors are entities that bring in drastic change in behaviour and expectation of existing entities, challenging traditional ideas and models be it in business, education or any other sector. Augmented reality, artificial intelligence, block chain and other advances in technology are at the fore-front of disrupting how we do business, market, customize, how we experience the physical versus the virtual world and much more. Netflix, WhatsApp, Zoom may be common household names now, but they have all disrupted their respective sectors - Netflix has changed our entertainment experience, WhatsApp has disrupted traditional telecommunication and Zoom is the new platform for meetings, socializing and more (Carter, 2015).

**Fig. 1:**
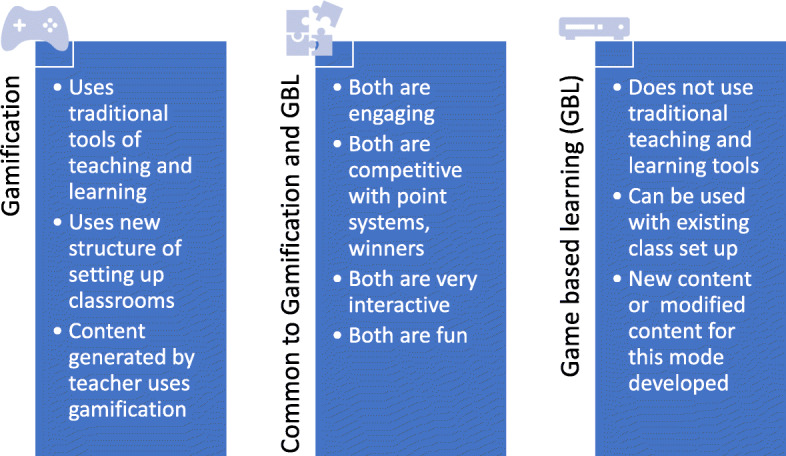
Similarities and differences between gamification and game-based learning (adapted from Baum, 2018)

One digital disruptor, gamification has gained popularity in education in recent years. Gamification, which is implementation of the rule of a game along with attributes like points, reward or punishment system into non-game settings, provides an opportunity to solve many problems in the area of education (Lee & Hammer, 2011; Smith, 2014; Packt, 2019). With the fundamentals of concepts taught using game-based learning strategies, gamification will make the experience fun, visual and adventurous (Baum, 2018). However, before we jump to discussing gamification, it is important to note that gamification and game-based learning are not one and the same. Game based learning (GBL) is often used as a strategy to enhance student learning experience and removes from traditional learning by focusing on “performance before competence” which, according to Gee (2012), is much more effective as a learning approach. As seen in the Fig. 1, there are differences between gamification and GBL as strategies to learning; that is, gamification is using some elements of gaming such as rewards systems and applying them to the traditional modules whereas GBL is replacing the traditional tools.

But just as important is to highlight and of consequence to this study is the similarity between the two as shown in the figure above - used rightly, these strategies can be engaging, interactive, competitive, and fun.

Gamification and GBL can positively impact on students’ emotional experiences which could be very useful for inculcating students on the correct attitude and behaviour in various scenarios. Gamification and GBL projects offer students the opportunity to experiment with rules, emotions, and social roles. Games also provide multiple routes to success, allowing students to take responsibility for their own decisions and actions (Locke & Latham, 1990). Games invoke a wide range of powerful emotions, from curiosity to joy or even frustration (Lazzaro, 2004). Games also provide positive emotional experiences, such as optimism and pride. They also help students to overcome negative emotions (McGonigal, 2011).

World Government Summit and Oxford Analytica (2016) report highlighted the benefits of using gamification in education to produce personalized learning for students. Literature has further shown how successful gamification and GBL as strategies to teach students moral values. A recent study in the UAE has demonstrated just such a success. The Moral Education Programme (MEP) led by the Education Affairs Office of the Crown Prince Court in Abu Dhabi showed how 90% of the student samples that were tested had over 70% success rate in learning universal values (Abu Dhabi Education Guide, 2019). In fact, gamification and GBL as tools to engage students and train staff has gained some momentum among AI researchers, academics and institutions in recent years through the works of Amanda White from the University of Technology (Sydney) and Sarah Eaton from the University of Calgary (Canada). Amanda White has created a board game (White, 2020) while Sarah Eaton has documented her experience of gamifying an AI workshop for staff (Eaton, 2019), and True North/Carnegie Mellon University’s Entertainment Technology Center developing a scenario-based game called A Fine Line (True North, 2016).

Based on review of existing literature, there remains limited scientific research and focus on gamification and GBL in teaching AI and a gap exists in extensively exploring these strategies in the academic integrity sector. The next sections record the objective and purpose of the gamification working group and this paper, paving path for academics and researchers who may be interested to use GBL and gamification in transforming their academic integrity modules.

It should also be noted that some authors seem to have missed out on explaining the relation between artificial intelligence to gamified education (Maud et al., 2018). Artificial intelligence refers to machines that can demonstrate intelligence that may be typically associated with humans (Shubhendu & Vijay, 2013). Such systems “(...) help people anticipate problems or deal with issues as they come up” (West, 2018), and will help gamified education to learn about the intentions of the user who is a student in this case, their moods, emotions and path they have taken with respect to various scenarios they have encountered in the past (Jantke, 2014). Artificial intelligence makes a digital system easy to learn and personalise it while gamification makes it attractive and playful (Biju, 2013). This has also been seen in various case studies like ( Jantke and Torsten, 2015) and will be taken into consideration in the design and development phase of the project.

## Gamification working group at ENAI

European Network for Academic Integrity (ENAI), as a part of its integrity enhancing activities has established a working group for promoting gamification of academic integrity. The working group aims to explore gamification as a tool to enhance engagement and commitment of academic stakeholders (students, staff, faculty members, management, and parents) towards teaching and learning of academic integrity values, thus working towards incorporating a proactive action in building a culture of integrity.

## Objective of this paper

The group met several times via video conferencing to discuss the vision and goal of the group and to proceed with fulfilling the objective of the working group. In the first instance, the group decided to look at one aspect of academic integrity as a pilot. The scope was “contract cheating” due to the proliferation of this type of misconduct and the far-reaching instances recorded.

Once decided, the next step was to divide the pilot study into phases. This paper records the results and findings from phase one of the study.

In this study, the group aims to look at proposing steps we are currently using to develop storyboards of scenarios for the first phase of the project which were presented at the 6th International Conference Plagiarism Across Europe and Beyond 2020 held virtually in Dubai as a workshop. The study also presents updated findings and scenarios drawn from the workshop conducted and audience feedback in the following sections.

## Methodology

Game development is a multimillion-dollar industry that spends as much as it earns. Particularly, the educational technology (EdTech) development industry has somewhat of a monopoly such that EdTech companies seem to be the ones that develop such technology and academics seem to naturally expect them to (Hawkins, 2018). This expectation and project management issues such as project scope creep, overshooting deadlines and overusing resources feed the wheel, making such EdTech often unavailable to those who cannot afford them (Kim, 2017).

As educators driving this project, the working group aims to therefore research, analyse and develop the gamified modules in house, and look at an open-access model in line with ENAI”s free resource initiative to make the final products available to academics, students and institutions to use the products to support academic integrity value learning.

**Fig. 2:**
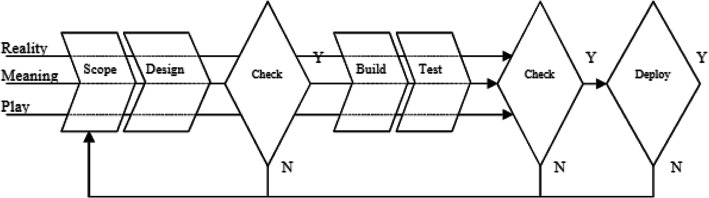
Triadic Game Design development model (Kortmann and Harteveld,  2009)

In order to develop the gamified academic integrity modules, an agile model of development will be used as it has become popular and supported by researchers and practitioners as an effective method (Kortmann & Harteveld 2009; Godoy & Barbosa, 2010). Duke & Geurts (2004) identified five steps commonly used in game design - setting the stage, clarifying the problem, designing the game, developing the game and implementation which are similar to the traditional software development stages, that is scope, design, build, test and deploy. Harteveld (2011) has posited that game development needs to take place in an iterative method to include users and taking into consideration feedback to go back and forth with every iteration, using the five stages of development commonly used for software development as mentioned above. In particular, we will be using an agile development model based on Kortmann & Harteveld’s Triadic Game Design (2009) as seen in Figure 2 below, which posits the TGD philosophy that state the game developers typically have to take into account the triad of reality (game connected to real world in terms of game subject, variables and definitions), meaning (how meaningful effect outside the game can be achieved) and play (selecting game elements like actors, rules, resources, challenges and competition) (Fig. 2).

Based on the game design stages, for phase one of this pilot stage of the project, the working group first discussed the aim of the gamification, the scope of the pilot study (contract cheating) and learning outcomes expected from the modules developed for this scope. Moving to the design step, as part of the practice to become competent, the group used brainstorming to identify scenarios in contract cheating as a pilot test case that students playing the game would engage with. Scenarios are narrative descriptions that provide details of the plot and individual scenes (Kahn & Wiener, 1967).

Once identified, the scenario was used to determine natural language script to help develop a conversational gameplay, “attempting to reach a specific outcome through a series of conversational “moves” (Lessard, 2016). Natural Language Script is a method used in software development process capturing “real conversation where a very wide spectrum of moves is available … [and] players can devise and perform their avatar’s utterances at the most fine-grained level - choosing wording, tone, accent, etc” (Lessard, 2016). The scripts have not been included in this paper due to constraints of space but can be produced upon request.

Based on the natural language script, a mind map was developed that recorded the natural progression based on identified options for the avatar.

**Fig. 3:**
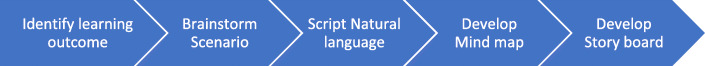
Steps to complete objective of this study - Phase One - Design, Pilot Study, Gamification of AI

Finally, based on the mind map, the group was able to create a storyboard. Storyboarding is a process that allows efficient and simple ways to develop a game or even a teaching plan, visually (Pradhan, 2018). This allows development of a plan of action, delivery timeline, and identifying errors early (Pradhan, 2018).  These stages are illustrated in the Figure 3 below.

## Result

Using the above method, two scenarios were identified which were part of one working group member’s experience, representing different lengths, cases.

### Scenario one

The first scenario involved students admitting not to approach faculty or staff whom they saw as “authority figures” and instead using their mobile phones for easy access to information. This led to students often being presented with contract cheating sites, essay mill portals or academic social networks (such as Coursehero, Chegg, etc). Although students understood these may not have been the most ethical options to get answers, they felt these were “less scary” than talking to someone in a position of authority.

**Fig. 4:**
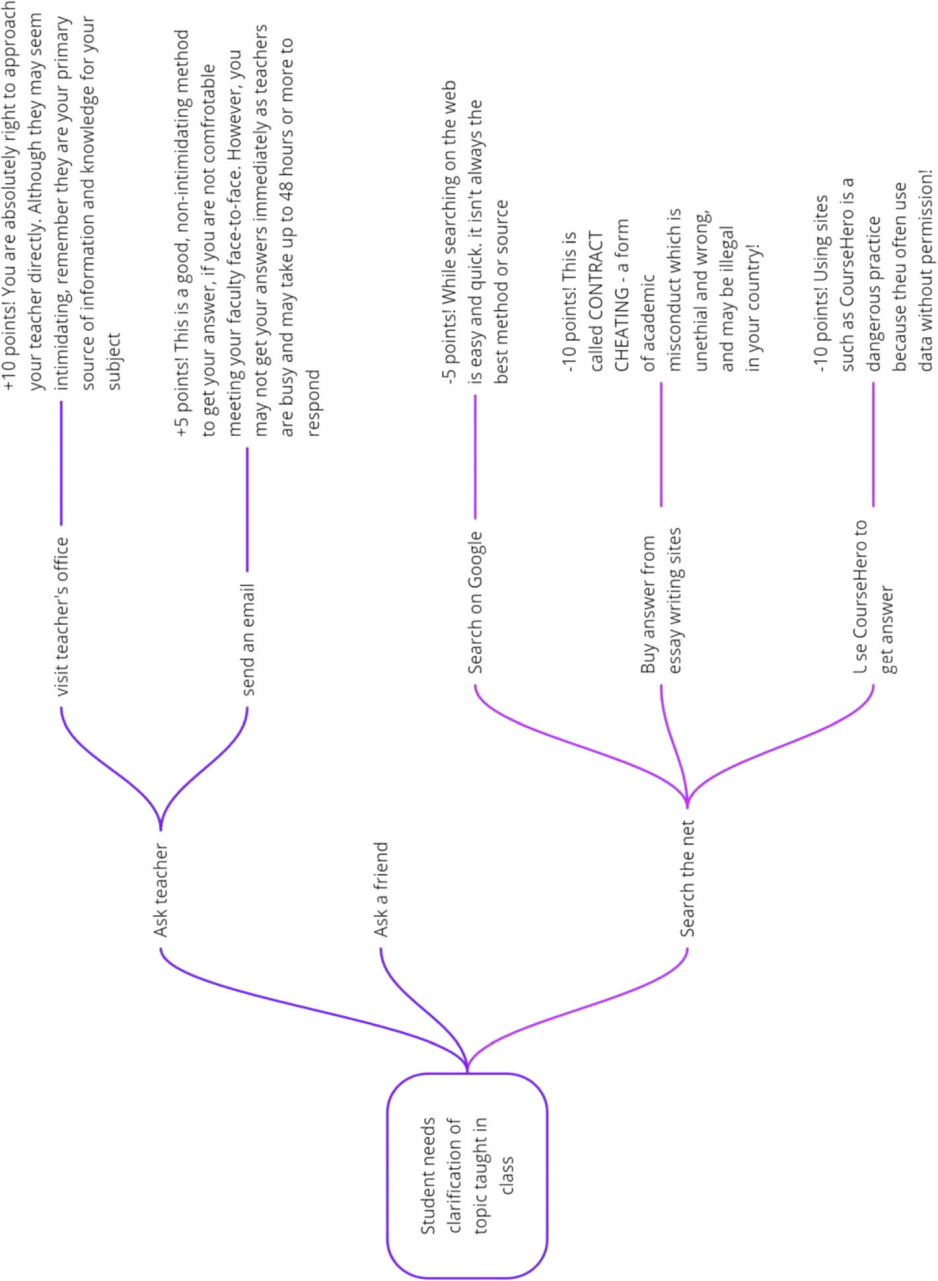
Mind map tracing Scenario 1

Based on this scenario, natural language script was developed which helped to trace out a mind map as shown in Fig. 4 using the online tool Miro.

**Fig. 5:**
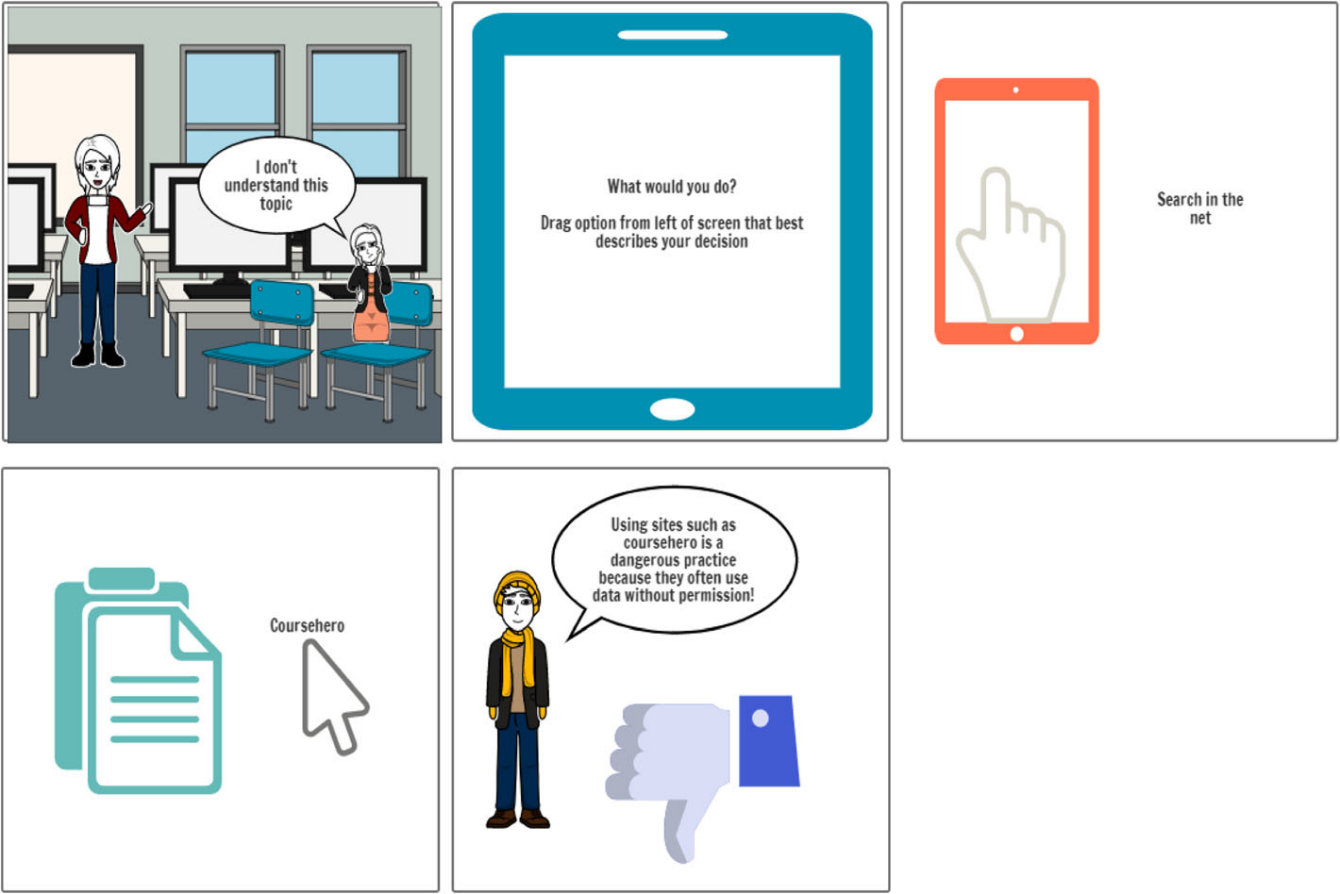
Storyboard for Scenario One

Once the mind map was created, it was used to create a storyboard (see Fig. 5).

### Scenario 2

Another scenario involved a story from one member of the working group’s 2019 finals experience. Note: all students’ names have been changed.

(Scene 1)

**Fig. 6:**
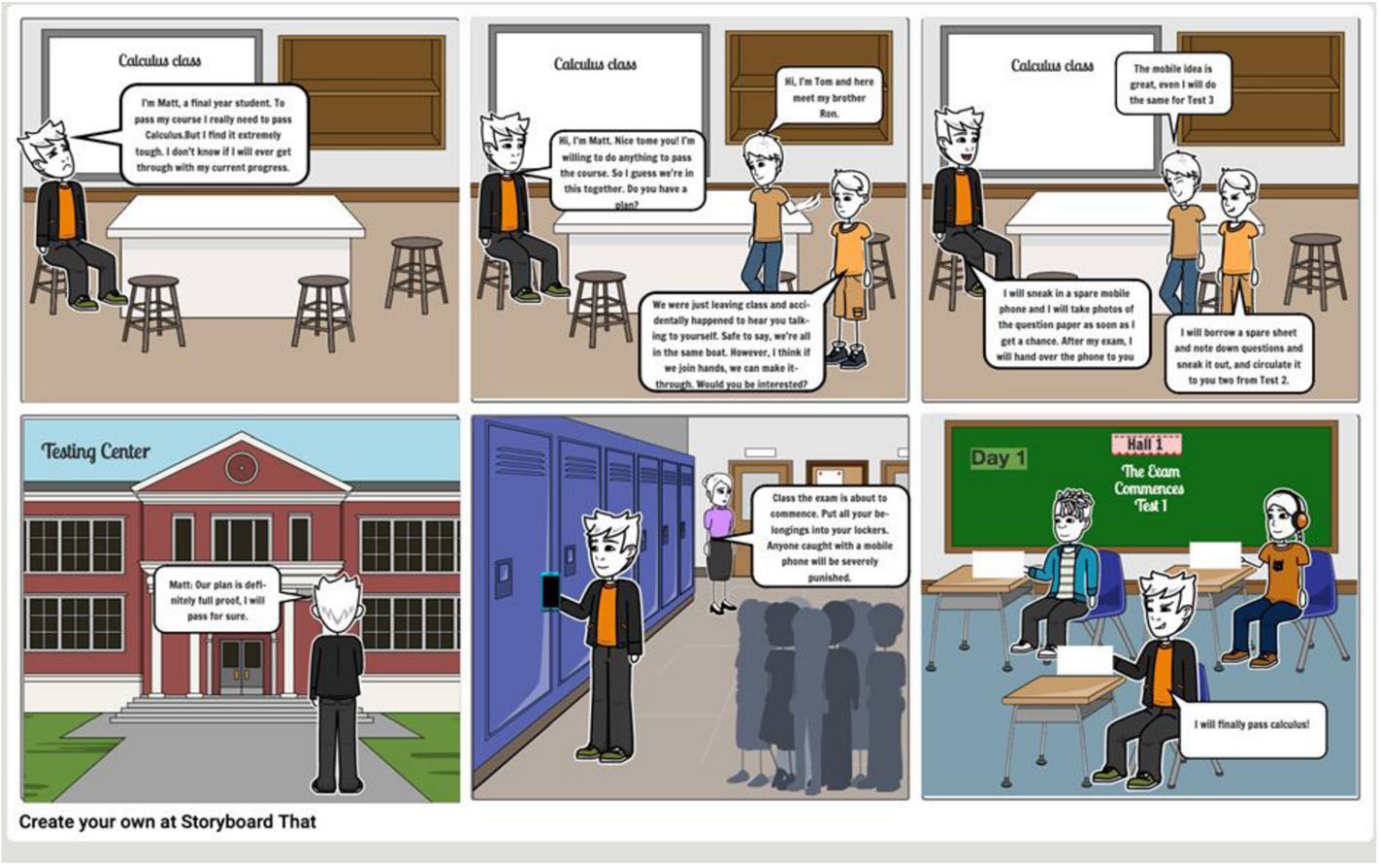
Storyboard of Scenario Two used in this study (Scene 1)

Student Matt was not doing well in Math. He was approached by two siblings, Tom and Rhonda who were also struggling in the class. Together, they decided that the only way they could pass the class was to arrange for the test materials to be shared among the three of them. They decided to rotate who would take the test and then share test answers with the group. Matt volunteered to take test 1. On test day he went into the Testing Center. At check-in, he removed his cell phone for the proctor/invigilator and placed it in a locker. He then started his test. See Figure 6 below

(Scene 2)

After sitting, he removed a hidden, second cell phone from under his shirt and proceeded to take pictures of the entire test. After the test, he showed the pictures to his friends, so they could study the test questions before taking the test. They even had the audacity to make cheat sheets and hid them in their clothes which they brought into their test.

**Fig. 7:**
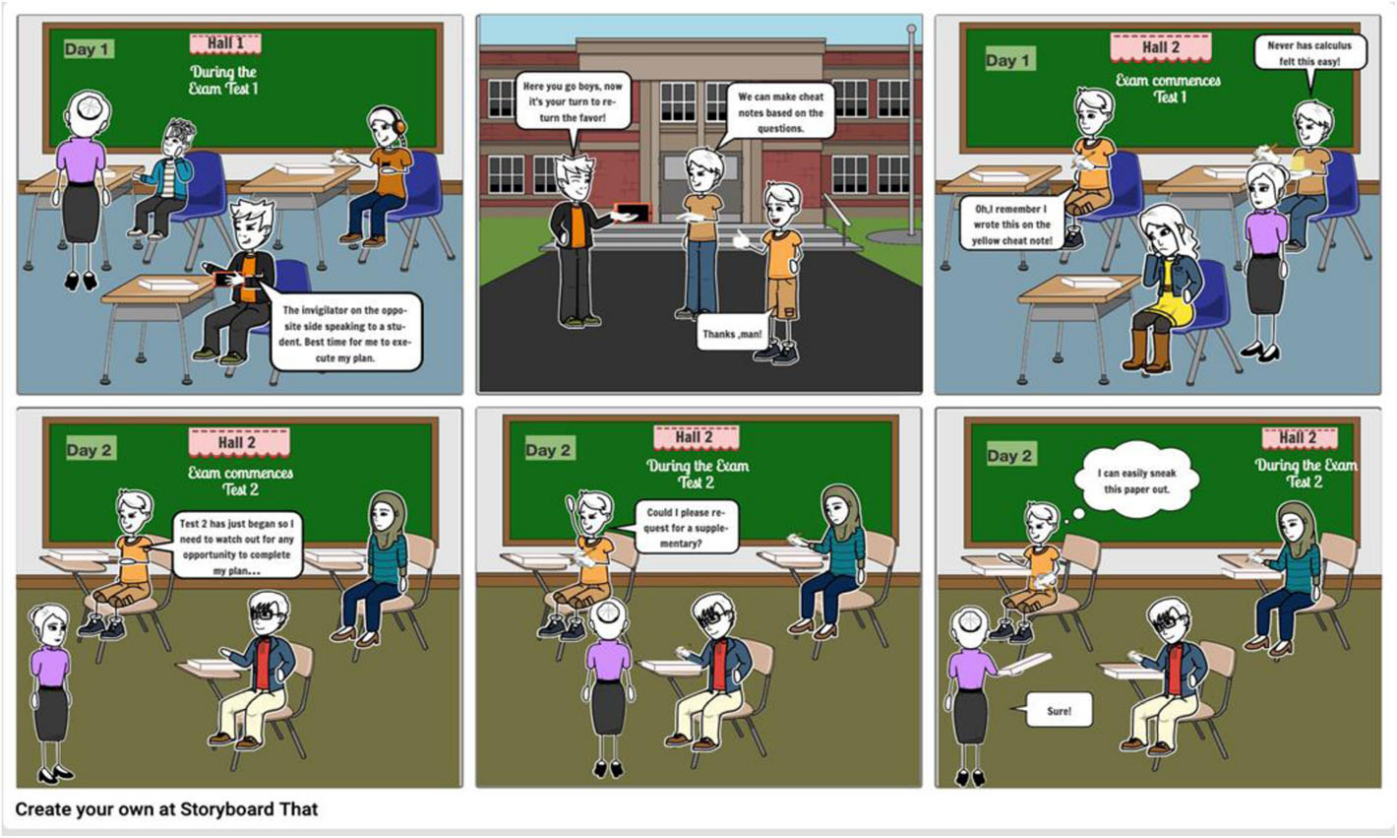
Storyboard of Scenario Two used in this study (Scene 2)

The pattern continued with Tom taking test 2. To change the routine, he copied the test questions onto scratch paper while in the Testing Center. During the test, he requested several additional pages of scratch paper from the invigilator and wrote his work on them. See Figure 7 below

(Scene 3)

Then, when finished testing, he slid the scratch paper with the test questions up his shirt sleeve. He stood and walked out of the Testing Center, handing his test and the scratch paper showing his work on the problems to the invigilator. The scratch paper up his sleeve had the full test copied onto it. This he snuck out of the Testing Center.. After the test, he shared the test questions with the other students.

Rhonda took test 3. Like Matt, she used her cell phone to take pictures of the test.

All three students were caught and issued both academic and institutional sanctions. Matt, due to prior offenses, was placed on probation and required to complete a 15-h course on academic integrity.

**Fig. 8:**
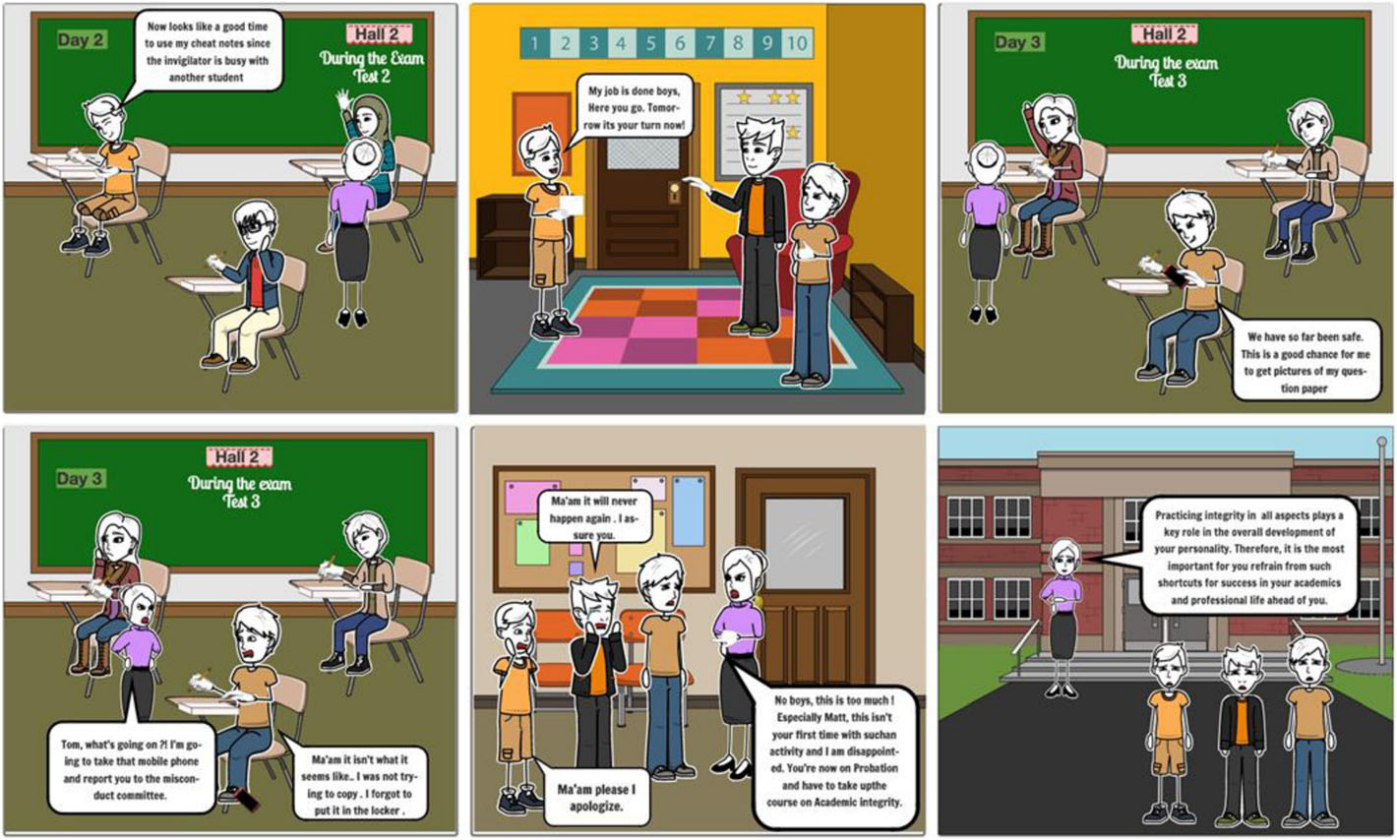
Storyboard of Scenario Two used in this study (Scene 3)

Based on the natural language scripting and mind map, the following storyboards were developed:

The above examples show how the project is identifying and creating storyboards for the gamification of integrity values and knowledge on contract cheating. This is the first phase of the project. See Figure 8 below

#### Next steps

The project is at this early phase where we are now developing storyboards from identifying possible scenarios to highlight. We were originally aiming to carry this work forward using the ENAI annual conference workshop by taking the audience through the step by step process of identifying scenarios and then developing storyboards. This would have helped us to ultimately produce sample storyboards as take-aways for the participants. This would have also aided us to add to the database of scenarios for the project’s sext stages. However, due to current Covid-19 related lock-down, this step-by-step interaction was not possible. Yet, we have captured the general feedback from the attendees which will help us to generate similar scenarios in future.

After developing an extensive, inclusive, and comprehensive database of such scenarios and transforming them into storyboards, the sessions will be piloted on focus groups of students to capture the effectiveness of the storyboards. Reflecting on the experiences from these pilot studies, any non-effective/duplicate scenarios will be eliminated. The project will then move to its implementation phase where we will develop the game on contract cheating. This is the future scope of the project.

#### Concluding remarks

Further discussion should consider how to design online games more effectively and how to integrate them into teaching and learning of academic integrity. The range of GBL and gamification have to be in accordance with the needs of the learners’ abilities and skills. There is a clear necessity for more rigorous research in understanding the efficacy of GBL and gamification and how to use these strategies appropriately in teaching academic integrity. In this process, it is worth exploring concepts of cultural preferences, native languages and personality and learner types. It goes without saying that one size does not fit all in anything in life and such stands true for higher education.

Exploration of affordances of GBL and gamification in teaching academic integrity are alternatives that may help students and educators alike, nevertheless it does require a careful consideration of a variety of factors that may be academic or personal. The rationale behind gamification of academic integrity modules is related to active involvement in processes of teaching and learning of two main stakeholders: students and educators; unlike current experiences when students are required to learn modules and then apply the knowledge without really having a say in the process and without taking any active roles.

This study has provided the first steps towards scientifically developing gamified learning modules on academic integrity, with specific focus on contract cheating for the first pilot stage of the project using gamification and game-based learning strategies. The study, by extension the working group’s overreaching purpose is not only to develop a gamified module and make it available to the stakeholders, but also to provide step-by-step simple ways to follow for any future academics and researchers who may wish to also look at GBL and gamification as strategies to enhance student learning and develop a culture of integrity proactively.

## Data Availability

All data is available upon request.

## Abbreviations

ENAI: European Network for Academic Integrity
QR: Quick response
GBL: Game based learning
AI: Academic integrity
UK: United Kingdom
MEP: Moral education programme
EdTech: Educational technology
